# Staffing Instability and Functional Decline in Long-Stay Nursing Home Residents with Dementia

**DOI:** 10.1093/geroni/igaf122.3655

**Published:** 2025-12-31

**Authors:** Talia Benheim, Christopher Santostefano, Fangli Geng

**Affiliations:** Brown University, Providence, Rhode Island, United States; Brown University, Providence, Rhode Island, United States; Brown University, Providence, Rhode Island, United States

## Abstract

Staffing instability in nursing homes may have adverse outcomes for residents with Alzheimer’s disease and related dementias (ADRD), yet most prior work has focused on average staffing levels. We linked Payroll Based Journal data to Minimum Data Set assessments from 15,828 facilities from 2018-2022 to examine associations between changes in staffing instability and care outcomes among long-stay residents with ADRD. The primary exposure was the quarterly number of “low outlier days” (LOD), defined as days with total direct care hours per resident at least 20% below the facility’s quarterly mean. We used an event study framework leveraging variation in the timing of changes in LOD, with facility and quarter fixed effects, adjusting for time-varying characteristics (overall staffing hours, staffing composition, mean census, resident case mix, resident COVID-19 cases), and weighting by the ADRD resident census. A 10-day increase in LOD was associated with a 0.5 percentage point (pp) increase in the share of residents with ADRD experiencing decline in Activities of Daily Living, a 0.3pp increase in worsened ability to move independently, a 0.2pp increase in significant weight loss, and a 0.1pp increase in pressure ulcers (all p<.001), but was not significantly associated with preventable hospitalizations or antipsychotic prescribing. Associations were largest for instability in certified nursing assistant (CNA) staffing, followed by licensed practical nurses and registered nurses, which aligns with CNAs’ roles in mobility and feeding assistance. These findings suggest that beyond increasing the quantity of staffing, reducing day-to-day instability should be a target for quality improvement.

